# Interventions to enhance the adoption of asthma self-management behaviour in the South Asian and African American population: a systematic review

**DOI:** 10.1038/s41533-017-0070-6

**Published:** 2018-02-15

**Authors:** Salina Ahmed, Liz Steed, Katherine Harris, Stephanie J. C. Taylor, Hilary Pinnock

**Affiliations:** 1 Asthma UK Centre for Applied Research, Blizard Institue Queen Mary University London, London, UK; 2 Asthma UK Centre for Applied Research, Usher Institute of Population Health Sciences and Informatics, The University of Edinburgh, Edinburgh, UK

## Abstract

South Asian and other minority communities suffer poorer asthma outcomes, have a higher rate of unscheduled care and benefit less from most existing self-management interventions when compared to the majority population. Possible reasons for these differences include failure to implement asthma self-management strategies, or that strategies implemented were inappropriate for their needs; alternatively, they may relate to the minority and/or lower socioeconomic status of these populations. We aimed to synthesise evidence from randomised controlled trials for asthma self-management in South Asian and Black populations from different sociocultural contexts, and identify barriers and facilitators to implementing self-management. We systematically searched eight electronic databases, and research registers, and manually searched relevant journals and reference lists of reviews. Seventeen trials met the inclusion criteria and were analysed narratively. We found two culturally targeted interventions compared to fifteen culturally modified interventions. Interventions used diverse self-management strategies; education formed a central component. Interventions in South Asian and African-American minority communities were less effective than interventions delivered in indigenous populations in South Asia, though the latter trials were at higher risk of bias. Education, with continuous professional support, was common to most interventions. Facilitators to asthma self-management included: ensuring culturally/linguistically appropriate education, adapting to learning styles, addressing daily stressors/social support and generic self-management strategies. In conclusion, when developing and evaluating self-management interventions aimed at different cultures, the influence of sociocultural contexts (including whether patients are from a minority or indigenous population) can be important for the conceptualisation of culture and customisation of self-management strategies.

## Introduction

South Asian communities, along with other minority populations, have poorer asthma outcomes, higher rates of hospital admission, greater risk of rehospitalisation and a higher death rate compared to majority white populations.^1–3^ Asthma self-management, consisting of education, written Personalised Asthma Action Plans (PAAPs) and regular reviews (supported self-management) is known to improve health outcomes, and is recommended in national and international guidelines.^4–6^ Despite hopes that self-management offers a potential solution to address preventable health inequalities,^1,5,7^ there are concerns that asthma self-management interventions have produced little or no positive improvements on health outcomes for South Asians or other minority populations, further widening the gap of asthma inequalities.^7–10^ Possible explanations for these variations include differences in health-seeking behaviours related to health beliefs and attitudes to mainstream medicine,^1,7,11^ environmental or lifestyle factors,^1,5,11,12^ poor healthcare access and the quality of asthma care provided to these communities.^13^ These factors may be driven by cultural diversity, by the experience of being a minority and/or by socioeconomic status (SES). Thus, the way in which self-management is accessed and delivered to these various populations, need to be explored, and self-management strategies may need to be developed for the target population’s culture, ethnicity, SES or other needs.^1,5,7^

There are distinctions between the way interventions can be made relevant to a population (see Table 1). ‘Culturally modified/adapted’ interventions, are developed for a majority population and then modified for use in other ethnic groups; the core content, however, is the same. ‘Culturally targeted’ interventions are developed from a bottom-up process that considers the shared characteristics and context of a cultural group before developing an intervention. Finally, bottom-up interventions that assess and are aimed at the unique cultural characteristics and dimensions of individuals within a cultural group, with individualised intervention delivery are known as ‘culturally tailored’.^14,15^ Culturally targeted or tailored interventions are generally suggested to be more effective than culturally modified interventions, though the evidence for this has focussed mainly on children,^14,16,17^ is limited or out-dated.^5,14,17–19^

**Table 1:** Definition of terms

Term	Definition	Examples
Culturally modified/adapted interventions^14^	Pre-existing generic interventions modified for the intention of being relevant to ethnic groups using various strategies, though the content is primarily the same	Language translation, and use of images and bilingual educators from a similar ethnicity as the target population
Culturally targeted interventions^15^	A bottom-up process which considers the shared characteristics and dimensions of collective individuals of a culture before developing an intervention, aimed at a group level	Religion
Culturally tailored interventions^15^	A bottom-up process which considers the unique cultural characteristics and dimensions of individuals within a cultural group before developing an intervention, aimed at individuals within a group	Level of religious identification or spirituality
‘Majority’ South Asians	Interventions from South Asian countries where the population forms a majority	South Asians in India
‘Minority’ South Asians; ‘Minority’ African Americans	Interventions from countries where the population forms a minority	South Asians in the UK or Canada; African Americans in the USA

Studies and clinical practice guidelines often indiscriminately apply findings from a majority population in a South Asian country, as relevant and applicable to South Asian minorities and majorities in other countries, despite differences in time and space of lived experiences and cultural shifts.^20,21^ Not only are the South Asian and Black population heterogeneous groups, but culture is fluid and continuously being shaped and reshaped across time and place, depending on an individual’s interaction with, and ability to respond to, the variability in their environment. Overlooking this ‘contextualisation’ may hinder adoption of self-management behaviour. Conversely, education aimed at cultural context enhances meaning, receptivity, relevance and processing of information by patients.^20,22,23^ Comprehension of a patient’s contextual realm offers a deeper understanding of the dynamic nature of cultural influences on self-management behaviour e.g., collective perceptions of asthma, familiarity with self-management and availability of, or access to, resources. This raises the question of whether poor asthma outcomes in ethnic minorities can be explained by their minority-status and/or by their relative social deprivation.^6,16,19,24–28^ These differences within a cultural group can influence the level of organisational and structural asthma inequalities faced by patients.^29^

This systematic review aims, in South Asian and Black communities (majority and minority populations), to (1) describe features of culturally relevant asthma self-management interventions, (2) synthesise the evidence for the effectiveness of interventions in different sociocultural contexts, and (3) identify barriers and facilitators to asthma self-management behaviour. We included interventions from South Asian countries where the population forms a majority (‘majority’ South Asian), and interventions from countries where the population forms a minority (‘minority’ South Asian; ‘minority’ African American) (see Table 1). We included studies of Black minority populations because our scoping work suggested that there was important literature, especially in African-American communities. This also allowed exploration of both the role of South Asian ethnicity, specifically versus the impact of minority/majority status on self-management outcomes.

## Results

### Characteristics of included trials

**Fig. 1:**
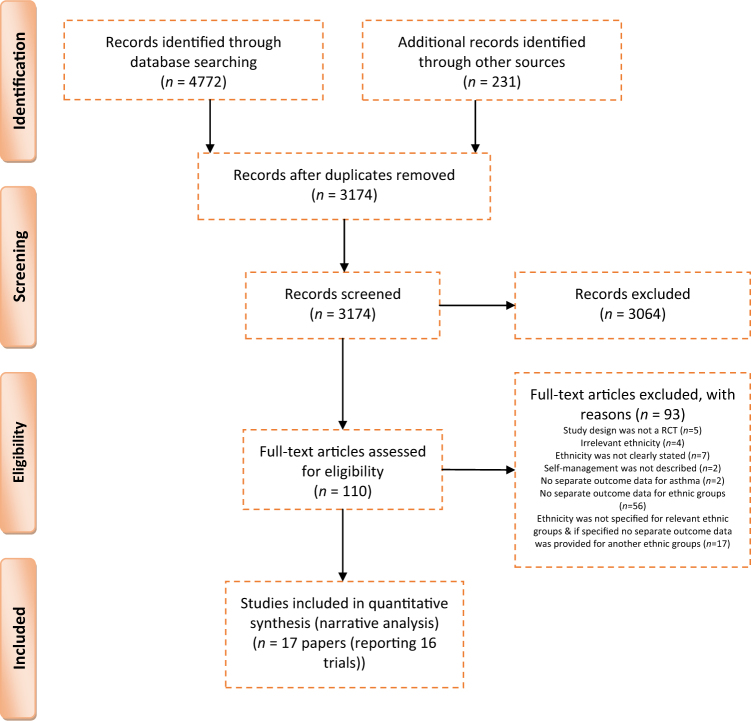
PRISMA flow diagram

From a total of 3174 citations, we included 17 papers (reporting 16 trials) (see Fig. 1). The randomised control trials (RCTs) were conducted between 1995 and 2016; four South Asian trials were from India (labelled ‘majority’ South Asian),^30–33^ four South Asian trials were from the UK^34–36^ and one from Canada^37^ (labelled ‘minority’ South Asian), and nine African-American trials were from the USA (labelled ‘minority’ African American)^26–34^ (see Table 2). The overall risk of bias within trials was uncertain,^30,33,37–41^ or high.^31,32,36,42–45^ Three trials had low risk^34,35,46^ (see Table 3).

**Table 2:** Overview of study characteristics of included trials

Study, Country	Population characteristics			Intervention characteristics				
	Aim	Ethnicity; Participants; Sample age; Sample size (*I*/*C*)	Study setting; SES/area	Intervention description/length	Control /other group descriptions	Delivery (ethnicity; language)	Mode of delivery	Modified; Targeted; Tailored
‘Majority’ South Asian trials								
Agrawal^30^ India	Evaluated efficacy of PAAPSs for asthma control	Indian; Patients; parents; 2–12; 60 (32/28)	Tertiary (university clinic)	Education; sessions, training including on asthma symptom diary and peak flow measurements	No PAAP, standard asthma therapy and education	Trained physician; social scientist (-)	Individual; Written material	Modified
			–	PAAPs				
				Asthma therapy/not stated				
Behera^31^ India	Assessed patient knowledge of self-care needs and develop/evaluate a self-care manual	Indian	Tertiary (outpatient university clinic)	Education-booklet in Hindi (included a PAAP)	No specific instructions/pilot study used to develop booklet in Hindi (*n* = 45)	Not stated (Hindi)	Written material	Targeted
		Patients; 18–60; 523 (260/263)	–	Booklet evaluation/not stated			Other methods not stated	
Ghosh^32^ India	Assessed the impact of self-management education and training on health status and resource use	*Indian*	Tertiary (university clinic)	Education; sessions, training, written instructions, audio-visual aids, role models, group/scenario discussions	Regular care e.g. drug administration	Trained social scientist (-)	Group;	Modified
		Patients; Parents; 10–45; 276 (140/136)	–	Daily diary (included symptom assessment and financial workbook)			Written material	
				Asthma therapy				
				*PAAPs*/four 2 hour sessions				
Shanmugam^33^ India	Provided pharmaceutical care through partnership of pharmacists and patients for good asthma control	Indian	Tertiary (university hospital)	Education; sessions, asthma care diary in English and Tamil (including leaflet), PAAP and symptom log sheet	No pharmaceutical care	Not stated (English and Tamil)	Written material;	Modified
		Patients Age; −; 66 (33/33)	–	Medication counselling/not stated			Other methods not stated	
‘Minority’ South Asians trials								
Griffiths^34^ UK	Tested whether specialist nurses across ethnically diverse and deprived areas reduce unscheduled care	South Asians (mostly Bangladeshi) White Caucasians, Other (Black/African Caribbean/Other)	Primary/secondary (out-of-hours GP service/hospital)	Education; training based on guidelines, nurse review with advice	Usual care; single nurse visits to discuss asthma guidelines and check inhaler technique	Trained nurse specialists (partially; PAAPs explained in Sylheti)	Individual;	Modified
				PAAP explained in English and Sylheti			Written material;	
				Ongoing clinical support for professionals on computer prompts				
		Patients; 4–60; 164 (95/69)	Deprived/urban	Peak flow meters provided			Telephone	
				Oral corticosteroids/2 one hour visits for GP practices; 194 days				
Griffiths^35^ UK	Tested whether culturally specific education programmes adapted from USA interventions reduce unscheduled care	South Asians (Bangladeshi, Pakistani, Indian, Sri Lankan)	Primary (GP)	Education; session including PAAP, nurse follow-ups to book appointments (CDSMP), research training with video based on guidelines, South Asian actors and manualised programme (PACE)/PACE; two seminars; CDSMP; 2-hour session	Usual care; nurse delivered standardised consultation. No PAAP/follow-up appointments provided	PACE; Nurse specialists, Academic GPs CDSMP; Trained nurse specialists (South Asians)	Group;	Modified
		Patients; Primary/secondary care clinicians; 3 and above; 375 (183/192)	Deprived/urban				Video/DVD;	
							Written material	
Moudgil^36^ UK	Tested whether bilingual education of treatment optimisation and follow-up reduce urgent healthcare and improve quality of life	South Asian (mainly Indian and Pakistani), White European	Primary (GP)	Education; community sessions delivered in South Asian languages including written literature, education follow-up	Usual asthma care follow-up	Trained GP (South Asian)	Individual;	Modified
		Patients; GP; 11–59; 344 (171/173)	Low or medium deprivation/urban	Booklet including PAAP (based on BTS guidelines) and peak flow measurements			Written material	
				GP trained on prescribing, optimal treatment, knowledge and medication				
				Peak flow meter provided				
				Asthma therapy/40 minutes				
Poureslami^37^ Canada	Explored the effectiveness of different culturally relevant information formats and impact on self-management	South Asians (Indian Punjabi), Chinese	Other/tertiary (home, university clinic)-	Education; videos (physician-led, community and physician-led/community combination)	Pictorial pamphlet in either Mandarin, Cantonese or Punjabi	Research facilitators (South Asian)	Group/video	Targeted
		Patients; 21 and above; 45 (33/12)		Peak flow meter	/Co-development of intervention (*n* = 35); focus group sessions (*n* = 40)			
				PAAPs/1 month				
‘Minority’ African American trials								
Blixen^42^ USA	Tested feasibility of a culturally appropriate in-patient education programme for hospitalisation	African Americans	Tertiary (hospital)	Education; sessions and video, asthma workbook using African-American images, references to famous celebrities, written education posted as follow-up	Received usual care	Trained nurse (Not stated)	Individual;	Modified
		Patients; 8–50; 28 (14/14)	–	Peak flow meter; MDI spacer provided			Video/DVD;	
				Toll free numbers for asthma organisations/Three 1-hour sessions			Written material	
Fisher^38^ USA	Tested community-based intervention to improve asthma awareness, attitudes, management practices and reduce acute care	African Americans, White Caucasians, Others	Other (community, school)	Education; promotion campaigns, sessions, training residents to support patients in school and community/12 months	Four areas in the same location with similar SES characteristics	Trained university staff/residents (African American)	Group;	Modified
		Patients; parents 5–14; 249 (100/149)	Low income				Individual	
Fisher^46^ USA	Tested whether community health workers can reach low-income parents of hospitalised children and to reduce rehospitalisation	African American	Other/secondary (community, hospital)	Education; sessions by asthma coach based on guidelines and parental support contacts/meetings for readiness to change, training for asthma coaches (including PAAPs)/2 years	Usual care; inpatient education and discharge planning with PAAP, a suggested follow-up primary care within 1 week of discharge	Nurse,	Individual;	Modified
		Parents; African-American Coaches; 2–8; 191 (97/94)	Low income/urban			psychologist,	Group;	
						three trained coaches (African American)	Telephone	
Ford^43^ USA	Reanalysed an education programme that assessed the effects on asthma outcomes	African Americans	Secondary (emergency department)	Education; sessions and follow-ups, handout, mailed sessions for non-attenders	Received no intervention	Trained healthcare professionals and nurses (not stated)	Group;	Modified
		Patients; 18–70; 241 (119/122)	Urban and rural	Visual medical card			Written material	
				Wallet sized card (with medication list, dose, frequency)				
				Placebo inhaler to practice/3 sessions				
Keslo^39^ USA	Provided major long-term therapeutic intervention and intensive education	African Americans	Secondary /tertiary (emergency department/university clinic)	Education; sessions based on NIH guidelines, Follow-up clinics	Usual care	Pharmacy researcher, pulmonologist (not stated)	Individual;	Modified
		Patients; 18 and above; 52 (30/22)	Low; deprived				Telephone;	
				Education booklet (including diary card for measurements and 1-page summary of asthma prevention, medications, triggers and peak flow meter product literature)			Written material	
				Asthma therapy for ICS				
				Peak flow meter (colour-coded stickers), inhaled b-agonist and aero chamber provided/1-hour session				
Keslo^44^ USA	Tested if a long‐term management programme (emphasising ICS and patient education), would improve outcomes	African Americans	Tertiary (university based clinic)	Education; session	Usual care from local physicians	Pharmacy researcher (not stated)	Individual;	Modified
		Patients; 18 and above; 39 (21/18)	Low; working and middle-class college students	Educational booklet			Group;	
				Written instructions on asthma crisis management			Written material	
				Asthma therapy and peak flow meter (colour-coded stickers), MDI and other medications				
				Follow-up clinics (including diary)/2 years				
Velsor-Friedrich^40^ USA	Tested the effect of a school-based education programme (Open Airways) on the psychosocial outcomes	African Americans	Other	Education; sessions/2 weeks, six 45 minute sessions per week	Usual care; participated in the Open Airways programme after intervention	Academic professor, nurse	Group	Modified
		Patients; 8–13; 102 (40/62)	(Eight public primary school with nurse clinics)			(-)		
			Low/Urban					
Velsor-Friedrich^41^ USA	An extension of the study above (Velsor-Friedrich 2004): tested a two-part school-based education programme	African Americans	Other (eight public primary schools with nurse clinics)	Education-sessions (as above)	As above and all students received a PAAP	Academic professor, academic nurse(-)	Individual; Written material	Modified
		Patients; 8–13; 52 (28/24)	Low/urban	A further 5-month visit with nurse where education information was reinforced, a packet of asthma information reviewed if needed, PAAPs adjusted, clinical assessment on medication and peak flow monitoring/7 weeks, 45 minute sessions, once per week				
Velsor-Friedrich^45^ USA	Evaluated efficacy of a school-based asthma education program on psychosocial & health outcomes	African Americans	Other (5 secondary schools)	Education; sessions, coping skills training including role-playing & technology use (with a booster session as follow-up)	Routine education	Clinician,	Individual; Group	Modified
		Patients; 13–19; 137 (74/63)	Low	Nurse practitioner reinforcement & clinic visit		nurse,		
				Provided MDI, hydro fluoroalkane & static free chamber		clinical psychologist		
				Peak flow diary		trained doctoral student		
				PAAP/Six 45 minute sessions over 6 weeks		(-)		

*Note*: Missing data obtained from authors is noted in italic in the table

**Table 3:** All included paper findings as reported and the decisions underpinning the harvest plots

Citation design, sample group/size and risk of bias score	Outcome categories, FU	Reported outcomes-values for intervention (*I*)/control (*C*) ^a^indicates the primary outcome (if stated)	Researcher’s interpretation for the harvest plot
Agrawal^30^ *n* = 60 children FU: 4 m	Clinical-unscheduled care, 4 m	Compared to controls, children in the intervention group had:	Illustrated as a consistent significant positive effect
		Fewer acute asthma events: *I*: 0.50 (SD 0.71) vs. 1.0 (SD 0.61); *p* = 0.02	
Overall risk of bias: Unclear	Clinical-asthma control, 4 m	Compared to controls, children in the intervention group had:	Illustrated as a consistent significant positive effect
		Improved symptom score: (from the symptom diary) *I*: 21.9 (SD 14.4) vs. *C*: 33.7 (SD 10.9); *p* = 0.0006	
		Fewer nocturnal awakenings: *I*: 1.75 nights/month (SD 1.30) vs. *C*: 3.25 (SD 1.20); *p* = 0.001	
		Reduced school absenteeism: *I*: 1.5 days/month (SD 1.4) vs. *C*: 2.54 (SD 1.79); *p* = 0.015	
	Process	Not assessed	–
	Behavioural	Not assessed	–
Behera^31^ CCT *n* = 523 adults	Clinical-unscheduled care, 1 yr	A reduction in hospital admissions is illustrated graphically (the authors state that there was a significant decrease in hospital admissions in the intervention group at FU compared to the control group)	Illustrated as a consistent significant positive effect
FU: 2 wks, 6 m, 1 yr Overall risk of bias: high	Clinical-asthma control, 2 wks, 6 m, 1 yr	Symptom scores decreased in both groups	Illustrated as a consistent significant positive effect
		*I*: Baseline: 18.14 (SD 41.23) vs. FU 1 yr: 12.61 (SD 28.66)	
		*C*: Baseline: 18.76 (SD 42.64) vs. FU 1 yr: 10.69 (SD 24.30)	
		Logistic regression: compared to the control group, more intervention group patients showed a significant improvement in symptom scores at 2 w, 6 m and 1 yr (*p* < 0.001)	
	Process, 2 wks, 6 m, 1 yr	Knowledge scores increased significantly in the intervention group and fell in the control group;	Illustrated as a consistent significant positive effect
		*I*: Baseline: 13.04 (SD 4.06) vs. FU 1 yr: 28.13 (SD 15.70); *p* = < 0.001 *C*: Baseline: 11.44 (SD 4.0) vs. FU 1 yr: 9.47 (SD 2.89); *p* = < 0.001	
		Logistic regression: Compared to the control group, more intervention group patients showed a significant increase in knowledge scores at 2 wks, 6 m and 1 yr (*p* < 0.001)	
	Behavioural, 2 wks, 6 m, 1 yr	Reported self-care in acute attacks showed no change in attitudes in either group, but significantly more patients in the intervention group adopted the recommended position (sitting, leaning forward) and practiced breathing exercises during an acute attack as compared to control patients	Illustrated as a significant positive effect but hatched to show inconsistency
Ghosh^32^ *n* = 276 adult, adolescent, children/parent	Clinical-unscheduled care, 1 yr (assessed by diary in months 4, 8 and 12)	Fewer total number of ED visits, but no between group difference in proportion with ED visit	Illustrated as positive but hatched to indicate inconsistency
		Number of ED visits in the 3-month diary: *I*: 11.6 (SD 16.2) vs. *C*: 21.8 (SD 25.0); *p* = 0.002	
		Proportion with ED visits in the 3-month diary: *I*: 42.9 vs. 50.0% (*p* = 0.117)	
		Number and duration of hospitalisations were both significantly reduced	
		Hospital days in the three diary months: *I*: 5.8 (SD 10.7) vs. 12.5 (SD 19.8); *p* = 0.016	
		Proportion hospitalised in the three diary months: *I*: 27.1 vs. *C*: 36.8%; *p* = 0.043	
FU: 4 m, 8 m, 1 yr Overall risk of bias: high	Clinical-asthma control, 1 yr (assessed by diary in months 4, 8 and 12)	Fewer productive days lost in the intervention group during the three diary months	Illustrated as a consistent significant positive effect
		Day lost: 17.6 (SD = 24.2)/34.1 (SD = 38.8); *p* = 0.003	
		PEFR was significantly improved in the intervention group relative to the control group;	
		Mean PEFR from diary cards *I*: 332 (SD 50.78) vs. 290 (SD 77.69); *p* = < 0.001	
	Process	Not assessed	**–**
	Behavioural	Not assessed	**–**
Shanmugam^33^ CCT *n* = 66	Clinical-unscheduled care	Not assessed	**–**
FU: 29 days Overall risk of bias: unclear	Clinical-asthma control, 29th day	Asthma control improved in the intervention group compared to the control group	Illustrated as a consistent significant positive effect
		Mean ACT score for each question was greater in the intervention group at FU: *p* < 0.05	
		(Overall mean ACT scores are not reported)	
		Lung function showed a greater increase in the intervention group compared with control	
		PEFR (L/min): Baseline: *I*: 282 (SD 95) vs. *C*: 265 (SD 93); FU: *I*: 336 (SD 88) vs. *C*: 268 (SD 85); *p* = < 0.05	
	Process	Not assessed	**–**
	Behavioural	Not assessed	**–**
Griffiths^34^ *n* = 44 practices/324 – (South Asians *I*: 95 *C*: 69 *n* = 164), adults, adolescents, children	Clinical-unscheduled care, 1 yr	[Note: these data are an *a priori* sub-group analysis]	Illustrated as a consistent no effect
		^a^Time to first unscheduled care effect on South Asians was not significant between intervention and control; South Asians HR 0.72, 0.48 to 1.09	
		^a^Proportion attending unscheduled asthma care: no between group differences in whole population. No data for South Asian sub-group, but authors state that ‘intervention effect was non-significant for other sub-group analysis’	
FU: 2 m, 9 m, 1 yr Overall risk of bias: low	Clinical-asthma control, 2 m, 1 yr	[Note: these data are an *a priori* sub-group analysis]	Illustrated as a consistent no effect
		Symptoms: no between group differences in whole population. No data for South Asian sub-group, but authors state that ‘intervention effect was not significant for other sub-group analysis’	
	Process	Not assessed	**–**
	Behavioural, 2 m, 1 yr	[Note: these data are an *a priori* sub-group analysis]	Illustrated as a consistent no effect
		Self-management behaviour: no between group differences in whole population. No data for South Asian sub-group, but authors state that ‘intervention effect was not significant for other sub-group analysis’	
Griffiths^35^ *n* = 84 practices/375 elders, adults, adolescents, children, primary and secondary care clinicians	Clinical-unscheduled care*I*: 171 days/*C*: 189 days*I*: 72 days/ *C*: 339 days1 yr	Unscheduled care: there was no between group difference in healthcare use	Illustrated as a consistent no effect
		^a^Time to first unscheduled contact FU: HR = 1.19 (0.92 to 1.53); *p* = 0.185	
		Proportion without unscheduled care FU: OR = 0.72 (0.45 to 1.16); *p* = 0.175	
		Time to first unscheduled primary care contact FU: HR = 1.20, 0.92 to 1.57 *p* = 0.177	
		Time to first routine review in primary care FU: HR = 2.22, 1.67 to 2.95 *p* = < 0.001	
		Corticosteroid prescriptions: There was no between group difference in steroid prescriptions	
		Steroids FU: *I*: 1.16 vs. 0.98 Adjusted incidence rate ratio: 1.14 (0.87–1.49)	
FU: 3 m, 1 yr Overall risk of bias: low	Clinical-asthma control, 3 m, 1 yr	Asthma control: there was no between group difference in symptom score	Illustrated as a consistent no effect
	Process, 3 m, 1 yr	Symptom score FU 1 yr: 9.9 (SD 5.0) vs. *C*: 10.1 (SD 4.2) AHR: −0.04 (−1.16 to 1.09); *p* = 0.949	
		Self-efficacy was improved at 3 m but not at 1 yr follow-up;	Illustrated as a consistent no effect. Another bar plotted to illustrate the 3 m finding—as a consistent significant positive effect
		At 3 months: *I*: 6.7 (2.1) vs. *C*: 6.3 (1.9) AHR: 0.44 (0.05 to 0.82); *p* = 0.027	
		At 12 months: *I*: 6.4 (1.8) vs. *C*: 6.3 (1.6) AHR: 0.25 (−0.13 to 0.63); *p* = 0.188	
	Behavioural	Not assessed	–
Moudgil^36^ *n* = 689 (White Europeans 345, Indian subcontinent 344); adults, adolescents, children	Clinical-unscheduled care, not stated ISC: *n* = 294 (*I*: 151 *C*: 143)	[Note: these data are an *a priori* sub-group analysis]	Illustrated as a consistent no effect
		Number of asthma events/episodes for South Asians: no between group differences	
		^a^Proportion with an admission. *I*: 5.3 vs. *C*: 6.3% OR 0.83 (0.28 to 2.44); *p* = 0.9081	
		Proportion with an A&E attendance. *I*: 1.4 vs. *C*: 4.0% OR 2.92 (0.52 to 21.2); *p* = 0.3184	
		Proportion with out-of-hours primary care. *I*: 2.8 vs. *C*: 2.6% FU: OR 0.95 (0.19 to 4.60); *p* = 1	
		Proportion with a GP consultation. *I*: 55 9 vs. 50.3%. OR 0.80 (0.49 to –1.30); *p* = 0.3971	
		Proportion with a steroid course. *I*: 20.3 vs. 19.9%. OR 0.97 (0.53 to 1.79); *p* = 1	
FU: 4 m, 8 m, 1 yr Overall risk of bias: High	Clinical-asthma control, 1 yr ISC *n* = 280	[Note: these data are an *a priori* sub-group analysis]	Illustrated as a consistent significant positive effect
		Quality of life in South Asians was significantly better in the intervention group	
		Change in AQLQ FU: *I*: 0.11 vs. −0.15. Between group mean difference 0.26 (0.17–0.36); *p* = < 0.001	
	Process	Not assessed	–
	Behavioural	Not assessed	–
Poureslami^37^ *n* = 92 (47 Chinese, 45 Punjabi); Adults	Process, 3 m, 6 mPunjabi *n* = 43	[Note: these data are an *a priori* sub-group analysis]	Insufficient data
		^a^Knowledge: no comparison data for intervention and control groups	
FU: 3 m, 6 m; 1 telephone survey interview Overall risk of bias: unclear	Behavioural, 3 m, 6 m	[Note: these data are an a priori sub-group analysis]	Insufficient data
	Punjabi *n* = 43	Understanding physician instructions; on ^a^medication and proper inhaler use skills: no comparison data for intervention and control groups	
Blixen^42^ *n* = 28, Adults	Clinical-unscheduled care, 3 m, 6 m	Healthcare use: no data provided, though stated as no significant between group differences	Illustrated as a consistent no effect
	Clinical-asthma control, 3 m, 6 m	Quality of life: There was no significant between group differences	Illustrated as a consistent no effect
		Overall AQOL score. FU 6 m: *I*: 4.59 (SD 1.48) vs. *C*: 4.43 (SD 1.52); *p* = 0.12	
FU: 3 m, 6 m Overall risk of bias: high	Process	Not assessed	–
	Behavioural, 3 m, 6 m	Self-management behaviours: no data, though stated as no-significant between group differences	Illustrated as a consistent no effect
Fisher^38^ *n* = 249 Adolescents, children, parents	Clinical-unscheduled care, Quarterly for 3 yrs	^a^Acute care: no data given (results illustrated graphically), though authors stated no significant between group differences in acute care (hospitalisations and ED attendances *p* = 0.35)	Illustrated as a consistent no effect
	Clinical-asthma control	Not assessed	–
FU: 3, 6, 9, 12, 16, 20, 24, 28, 32, 36 m Overall risk of bias: unclear	Process	Not assessed	–
	Behavioural, Every quarterly until 3 yrs	^a^Asthma management: no significant between group differences in the non-validated assessment of parent’s reported attitude about asthma and asthma management	Illustrated as a consistent no effect
		Attitudes about asthma FU: *I*: 2.34 vs. *C*: 2.24 (*p* = 0.35)	
		Appropriate thresholds for seeking help Baseline: *I*: 30 vs. *C*: 47%; FU: *I*: 51 vs. *C*: 53% *p* = 0.77	
Fisher^46^ *n* = 191/parents, coaches	Clinical-unscheduled care, 1 yr, 2 yr	^a^Hospitalisation Compared to controls, the intervention group had fewer hospitalisations;	Illustrated as a consistent significant positive effect
		Hospitalised at least once FU *I*: *n* = 35/96 (36.5%), 55 vs. *C*: 55/93 (59.1%); 95% CI (0.11–0.34); *p *= .002	
FU: 6, 12, 18, 24 m Overall risk of bias: low	Clinical-asthma control	Not assessed	–
	Process	Not assessed	–
	Behavioural	Not assessed	–
Ford^43^ *n* = 241 (African America*n* = 163, Caucasia*n* = 78)	Clinical-unscheduled care, 4 m, 8 m, 1 yr	^a^ED visits No impact [Note: these data are an *a priori* sub-group analysis]	Illustrated as a consistent no effect
		ED visits/year I: Baseline: 5.0 (SD 3.6) vs. FU 2.7 (SD 3.3); *C*: Baseline: 6.7 (SD 8.4) vs. FU: 4.8 (SD 6.8)	
		No between group comparisons reported	
	Clinical-asthma control, 4 m, 8 m, 1 yr	Limited days of activity No impact [Note: these data are an *a priori* sub-group analysis]	
		Days/person: *I*: Baseline: 20.6 (SD 25.4); FU: 18.7 (SD 36.8) *C*: Baseline: 27.8 (SD 33.4); FU: 27.9 (SD 55.7), no between group differences reported	
FU: 4 m, 8 m, 1 yr Overall risk of bias: high	Process, 1 yr	^a^Knowledge and beliefs: no effect [Note: these data are an *a priori* sub-group analysis]	Illustrated consistently no effect
		Mean scores *I*: Baseline: 14.1 (SD 2.9); FU: 14.6 (SD 3.2) *C*: Baseline: 14.3 (SD 2.3); FU: 14.7 (SD 2.3)	
		No between group differences reported	
	Behavioural	Not assessed	–
Keslo^39^ *n* = 52 adults	Clinical-unscheduled care, 1 yr	Unscheduled care: compared to controls, the intervention reduced ED visits but not hospitalisations	Illustrated as a significant positive effect but hatched to show inconsistency
		^a^Change in ED visits Baseline: *I*: 4.4 (SD 2.7) vs. *C*: 3.4 (SD 2.6); FU: *I*: 2.6 (SD 2.6 vs. *C*: 3.5 (SD 2.7) Between group difference *p* = < 0.01	
		Change in hospitalisations Baseline: *I*: 1.3 (SD 1.3) vs. *C*: 1.0 (SD 1.2); FU: *I*: 0.5 (SD 0.8) vs. *C*: 0.5 (SD 0.9) Between group difference *p* = 0.37	
FU: 1 yr, telephone every 2 wks to every 6 m Overall risk of bias: unclear	Clinical-asthma control	Not assessed	–
	Process, After intervention	No data reported for knowledge	Insufficient data
		No data reported for medicine treatments	
	Behavioural	Not assessed	–
Keslo^44^ *n* = 39, adults	Clinical-unscheduled care, 1 yr, 2 yr	Unscheduled care: Intervention group had a greater reduction in hospitalisations and ED visits	Illustrated as a consistent significant positive effect
		^a^Change in ED visits. Median (IQR) visits 2 years, *I*: 0 (0, 0) vs. *C*: 2 (1.5, 2); *p* = < 0.05	
		^a^Change in hospitalisations. Median (IQR) hospitalisations, *I*: 0 (0, 0) vs. *C*: 0.5 (0, 1); *p* = < 0.05	
FU: every month then every 2–3 m Overall risk of bias: High	Clinical-asthma control 6 m, 1 yr, 18, 2 yr	No control group data reported for quality of life, asthma bother or peak flows	Insufficient data
	Process, before and after intervention	No control group data reported for Knowledge control group	Insufficient data
		No control group data reported for medicine treatments control group	
	Behavioural	Not assessed	–
Velsor-Friedrich^40^ CCT *n* = 102, children	Clinical-unscheduled care, 2 wks, 5 m, 1 yr	Unscheduled care: the intervention group had significantly more unscheduled visits at 5 m and 1 yr	Illustrated as a consistent significant negative effect
		Medical visits at 5 m. Mean (SE) *I*: 0.12 (0.05) vs. *C*: 0.02 (0.04)	
		Medical visits at 1 yr. Mean (SE) *I*: 0.07 (0.03) vs. *C*: 0.00 (SD 0.02); *p* = 0.01	
FU: 2 wks, 5 m, 1 yr Overall risk of bias: unclear	Clinical-asthma control, 2 wks, 5 m, 1 yr	Symptom days: greater reduction in days with symptoms in intervention compared to control	Illustrated as a consistent positive effect but hatched to show inconsistency
		Symptom days at 5 m. Mean (SE). *I*: 2.15 (SE 0.30) vs. *C*: 1.42 (SE 0.21)	
		Symptom days at 1 yr. Mean (SE). *I*: 1.26 (SE 0.33) vs. *C*: 1.49 (SE 0.23); *p* = 0.047	
		PEFR: intervention group had greater increase in PEFR at both FU time-points	
		% increase in PEFR at 5 m. *I*: 2.9% (SE 2.0%) vs. *C*: 2.9% (SE 1.0%)	
		% increase in PEFR at 1 yr. *I*: 7.5% (2.0%) vs. *C*: 2.9% (SE 1.2%); *p* = 0.046	
		School absences: no between group difference in days absent from school	
		Days absent at 1 yr. *I*: 9.03 vs. *C*: 14.4 days	
	Process, 2 wks, 5 m, 1 yr	Knowledge, self-efficacy and self-esteem/motivation: no significant between group differences	Illustrated as a consistent no effect
		Asthma knowledge test at 5 m: *I*: 14.05 (SE 0.55) vs. *C*: 13.35 (SE 0.38)	
		Asthma belief survey at 5 m. *I*: 4.23 (SE 0.10) vs. *C*: 4.15 (SE 0.08)	
		Self-perception inventory at 5 m. *I*: 2.80 (SE 0.08) vs. *C*: 2.85 (SE 0.05)	
	Behavioural, 2 wks, 5 ms	Self-practice/asthma self-care: No significant between group differences	Illustrated as a consistent no effect
		Denyes self-care agency instrument at 5 m: 72.03 (SE 2.46) vs. 70.57 (SE 1.68)	
		Asthma self-care instrument at 5 m *I*: 68.87 (SE 2.89) vs. *C*:70.41 (SE 2.00)	
Velsor-Friedrich^41^ CCT *n* = 52, children	Clinical-unscheduled care, 2 wks, 5 m, 1 yr	Urgent medical visits (and medications): no significant between group differences at any time point	Illustrated as a consistent no effect
		Urgent doctor visits at 12 m. *I*: *n* = 4 (14%) vs. *C*: *n* = 5 (20%)	
		No data; some data on medicine use was provided	
FU: 2 wks, 5m, 1 yr, 2 yr Overall risk of bias: unclear	Clinical-asthma control, 2 wks, 5 m, 1 yr, 2 yr	Symptoms, PEFR and school absences: no significant between group differences at any time point	Illustrated as a consistent no effect
		Proportion with > 1 day with symptoms/2 wks at 1 yr. *I*: 14 (50%) vs. *C*: 13 (54%)	
		% increase in PEFR from baseline at 1 yr. *I*: 26.21% (SD 0.22) vs. *C*: 27.80% (SD 0.31)	
		Average days absent from school. *I*: 9.03 vs. *C*: 14.4	
	Process, 2 wk, 5 m, 12 m	Knowledge and self-efficacy: Intervention group had higher scores at all time-points, but neither group improved over time	Illustrated as a consistent positive effect but hatched to show inconsistency
		Asthma Knowledge: test at 1 yr. Adjusted mean *I*: 14.28 (SE 0.80) vs. *C*: 11.88 (SE 0.87); *p* = 0.03	
		Asthma belief scale at 1 yr. Adjusted mean *I*: 4.09 (SE 0.14) vs. *C*: 3.82 (SE 0.15); *p* = 0.01	
		Self-esteem: no significance between group differences at any time point	
		Self-perception inventory at 1 yr. Adjusted mean *I*: 2.71 (SE 0.08) vs. *C*: 2.78 (SE 0.10)	
	Behavioural, 2 wks, 5 m, 1 yr	Asthma self-care practice/general self-care: intervention group had higher scores at all time-points, but neither group improved over time	Illustrated as a consistent positive effect
		Denyes self-care agency instrument. *I*: 75.55 (SE 2.60) vs. 67.41 (SE 2.82); *p* = 0.01	
		General self-care. *I*: adjusted mean *I*: 72.99 (SE 3.26) vs. *C*: 63.75 (SE 3.53); *p* = 0.2	
Velsor-Friedrich^45^ RCT *n* = 137, adolescents	Clinical-Unscheduled care, 6 m, 12 m	Hospital visits: no significance between group differences *p* > 0.05 (no other data provided)	Illustrated as a consistent no effect
FU: 2 m, 6 m, 1 yr Overall risk of bias: high	Clinical-asthma control, 6 m, 1 yr	Symptoms reduced in both groups; no significant between group differences	Symptom takes priority. Illustrated as a consistent no effect
		PEFR: no significance between group differences	
		School absences reduced in both groups; no significant between group differences	
	Process, 6 m, 1 yr	Knowledge, self-efficacy improved in both groups; no significant between group differences	Illustrated as a consistent no effect
		Coping frequency/efficacy, no significance between group differences	
	Behavioural, 6 m, 1 yr	Self-care practice, no significance between group differences	Illustrated as a consistent no effect

For conflicting outcomes within a category, the decision process was dependent upon priority of evidence including:

• Defined primary outcomes in an adequately powered sample/sub-group analysis (for the latter we will consider a prior sub-group analysis)

• Outcomes measured using a validated instrument (as opposed to non-validated instruments)

• Outcomes that were clinically and statistically significant (e.g., achieved significance defined minimum clinically important difference)

• If doubts remain, the author’s interpretation was considered to provide context for the final decision

Note:

• For quality of life outcomes, we will use the overall score, if no overall score is stated the outcome will not be plotted

• Asthma related quality of life scales will be given priority (e.g., AQLQ) over generic quality of life scales (e.g., ED5D)

• For the clinical-asthma control category, symptoms will be a priority over other outcomes in the same category as it is a better indicator of asthma control

***Abbreviations;*** *FU* follow-up, *wks* weeks, *m* month, *yr* year, *RCT* randomised control trial, *CCT* clinical control trial, *ED* emergency department visits, *I* intervention, *C* control, *CI* confidence interval, *AQLQ* quality of life questionnaire, *AQ20* the airways questionnaire 20, *ACT* asthma control test, *F* F statistics, *AHR* adjusted hazard ratio, *HR* hazard ratio, *OR* odds ratio, *EES* estimated effect size, *PEFR* peak expiratory flow rate, *SD* standard deviation, *SE* standard error, *DF* degree of freedom, *p* *p*-values

*Participant characteristics*: The ‘majority’ population in the South Asian trials comprised of Indians,^30–33^ whereas ‘minority’ South Asian trials included Indians,^37^ and mixed subcultures (e.g., Bangladeshi, Pakistani, Indian or Sri Lankan).^34–36^ All Black population trials studied the African-American minority population in the USA.^38–46^ Most trials (fourteen studies) did not define ethnicity; only three ‘minority’ South Asian trials defined ethnicity according to self-identification or language spoken.^34,35,37^ All trials aimed interventions at asthma patients (whether this was children, adolescents, adults or elders).^30–46^ In addition, some trials also targeted parents,^30,32,38,46^ trained African-American coaches and/or residents,^38,46^ or healthcare professionals (clinicians and nurses).^30,32,34–36^

*Study setting*: All ‘majority’ South Asian trials were based in tertiary care hospitals.^30–33^ In contrast, ‘minority’ South Asian trials were conducted in primary care,^35,36^ or a combination of community, primary care and hospital (secondary/tertiary) settings.^34,37^ Similarly, the African-American trials were conducted in various settings: primary or secondary schools,^40,41,45^ tertiary care hospitals,^39,42^ emergency department^43^ and three trials used a combination of settings; community, school and hospital (secondary/tertiary).^38,44,46^

*Geographical area and socioeconomic status:* Among the ‘minority’ trials that specified the demographic location of patients, these were described as urban in six trials^34–36,40,41,46^; and one African-American trial was conducted in mixed urban and rural areas.^43^ Eight trials were described as from economically deprived or low-income areas,^34,35,38–41,45,46^ and two ‘minority’ trials (South Asian and African American) were conducted in low/middle-class areas.^36,44^

*Intervention characteristics*: Table 2 describes intervention characteristics. All interventions included patient education, though the approach, method of delivery and content varied. Examples included education-sessions or classes,^30,32,33,35,36,38–46^ training for patients,^30,32,34,35,38,45,46^ and healthcare professionals, coaches or residents,^30,32,34–36,38,46^ education in written,^31–33,35,39,43,44^ or video format,^35,37,42^ education in the form of social support,^46^ or a local education/promotional campaign.^38^ Twelve out of 17 interventions were delivered by healthcare professionals,^30,32,34–36,38–46^ five of whom were specifically trained for the project.^30,32,35,42,43^ Three interventions from minority countries were delivered in South Asian languages by healthcare professionals or research facilitators,^35–37^ two ‘majority’ South Asian trials had written materials in Hindi or Tamil,^30,33^ and two USA interventions were delivered by trained African American lay people or university staff who were residents in the community.^38,46^ Intervention duration ranged from 40 minutes to 1 year and follow-up lengths ranged from 1 month to 3 years (see Table 3 for details on the latter).

Strategies for reinforcing knowledge or self-management behaviours included follow-up classes,^36,45^ nurse clinics^34,35,39,41,44,45^ and written materials.^42,43^ Most trials described other intervention characteristics used alongside education,^30,32–46^ including the use of written PAAPs in all South Asian trials (majority and minority)^30–37^ and some African-American trials,^41,45,46^ provision of emergency oral corticosteroid courses,^34^ asthma medication/therapy,^30,32,34,36,39,42,44,45^ placebo inhalers to practice technique,^43^ asthma diary/workbook,^30,32,33,42^ peak flow monitoring,^30,34,36,37,39,41,42,44,45^ medication counselling^33^ and access to free asthma organisation helplines.^42^ In seven trials, intervention strategies were based on specific guidelines, e.g., National Institutes of Health, National Heart Lung and Blood Institute, Global Initiative for Asthma (GINA) and Scottish Intercollegiate Guideline Network (SIGN).^33–36,39,44,46^ Usual care for the control groups varied,^30–36,39–44,46^ including illustrative leaflets,^37^ routine education classes,^45^ and recruiting similar neighbourhood areas to the intervention sites.^38^

*(1) Features of culturally relevant interventions*. In line with our definition and that in previous literature,^14,15^ we did not find any culturally tailored interventions, and only two of seventeen trials evaluated culturally targeted interventions.^31,37^ Behera et al.^31^ (‘majority’ South Asian trial at high risk of bias) provided a targeted written self-care booklet in Hindi (including a PAAP) developed collaboratively from patient knowledge, relevant literature and expert advice. Poureslami et al.^37^ (‘minority’ South Asian trial at unclear risk of bias) developed educational videos in collaboration with community members and healthcare professionals. The educational videos included three intervention possibilities (i.e., scientific knowledge, community opinions/narratives or a combination of both), that incorporated cultural beliefs and attitudes, e.g., cultural gestures, humour, storytelling and social interaction styles appropriate for Punjabi Indians. The aim was to facilitate patients’ trust in the community member and/or clinician who delivered the intervention.^37^ Both interventions were piloted in focus groups to improve clarity, relevance and acceptability and were refined before evaluation. These trials were not classified as culturally tailored because they were delivered to the specified cultural group without distinguishing or measuring individual cultural differences within that group.^31,37^

Both trials significantly improved knowledge. Poureslami et al.^37^ improved adherence to physician instructions on medication and inhaler use, and Behera et al.^31^ reported reduced symptoms, hospital admissions and use of breathing exercises during acute attacks. Although, the former trial achieved significant findings on all outcomes for Punjabi Indians, the Chinese population (who were studied as a parallel group with their own culturally targeted intervention) performed even better. The authors considered that this may be related to participant demographics; the Punjabi Indians were older and less educated than the Chinese community.^37^

In contrast, 15 out of 17 interventions were found to be culturally modified.^30,32–36,38–46^ They used strategies such as adapting existing interventions or materials for the target ethnic group,^32,35,39,44^ e.g., an African-American training video was re-recorded with South Asian actors,^35^ and ethnically relevant images were used such as African-American celebrities.^34,35,42^ Other studies applied interventions to several ethnic groups without considering cultural differences; thus, providing written or oral education (e.g., classes, PAAPs and workbooks) translated from English to the target participant language or using bilingual educators, without adjusting intervention content.^33–36^ However, the distinction between modified, tailored and targeted interventions is not clear-cut. Both culturally targeted interventions also incorporated some modified components,^31,37^ e.g., adaptation of language in PAAPs to meet the target population needs.^31^

**Fig. 2:**
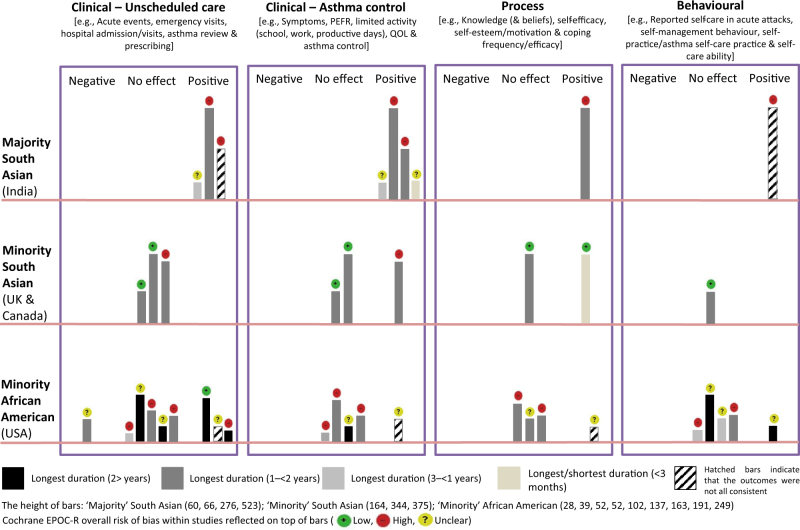
Harvest plots illustrating the effectiveness on clinical, process and behavioural outcomes of self-management interventions across different ethnic groups and social contexts. To determine the overall effectiveness of trials, plots were placed under each category (unscheduled care, asthma control, process or behavioural), according to whether findings were positive (i.e., interventions, which were significantly effective in the intervention group), negative (i.e., interventions, which were significantly effective in the control group), or outcomes that had no impact between groups.^50^ The colours of the plots in the graph represent the study length (long and/or short), the height of the bars represent the sample size and the icon on the top of the bars represent the overall risk of bias within studies

*(2) Effectiveness of interventions in different sociocultural contexts.* In the harvest plot (Fig. 2 and Table 3), the four outcome categories (i.e., unscheduled care, asthma control, process and behavioural), are plotted for the three ethnic groups, ‘majority’ South Asian, ‘minority’ South Asian and ‘minority’ African American.^47^ The harvest plots show that the interventions in the ‘majority’ South Asian trials were effective, though notably they were all based in tertiary care settings potentially serving a relatively severe asthma population (thus with greater potential for improvement).^30–33^ In addition, risk of bias, was either high,^31,32^ or unclear,^30,33^ and two of these trials had short follow-up periods (1 and 4 months).^30,33^

In contrast, trial outcomes from studies involving both ‘minority’ communities were inconsistent, though more trials were at a low risk of bias,^34,35,46^ in contrast to ‘majority’ trials. In the ‘minority’ South Asian trials, most of the outcomes did not show significant benefit.^34–36^ The exceptions were improved quality of life in a trial at high risk of bias,^36^ and in another study improved self-efficacy at 3 months, which was not sustained at 12 months.^35^ Similarly, in ‘minority’ African-American trials (all but one were at high or unclear risk of bias),^46^ most interventions were ineffective,^38,40–43,45^ or inconsistent.^39–41^ In addition, one trial at unclear risk of bias had a *negative* impact on unscheduled care.^45^ Three trials had positive outcomes (unscheduled care and behavioural),^41,44,46^ of which one trial was at a low risk of bias.^46^

**Fig. 3:**
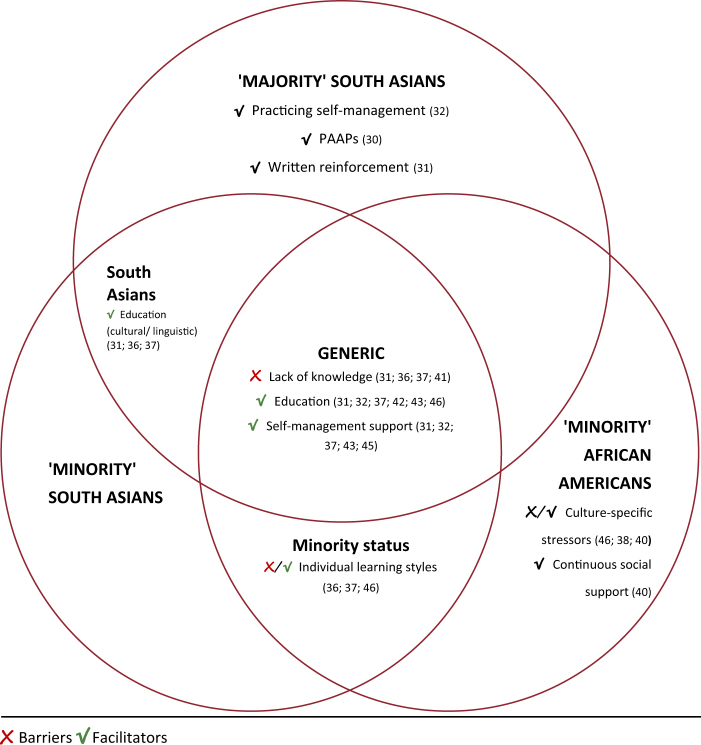
Summary of identified barriers and facilitators to asthma self-management in interventions across different groups

*(3) Identified barriers and facilitators to self-management in included trials.*A range of barriers and facilitators to asthma self-management were identified and differentiated according to ethnicity and sociocultural context (Illustrated in Fig. 3). Key findings were that:

- Across both ethnic groups and all social contexts, barriers included insufficient knowledge and understanding of asthma and related factors^31,36,37,43^; facilitators included providing self-management education,^31,32,37,39,44,45^ and support from healthcare professionals (with continuity of care).^31,32,37,41,44^
- In ‘minority’ trials, even though language barriers were accounted for,^36,37^ a barrier identified for South Asians, was insufficient consideration of individual learning styles related to age,^36,37^ gender^36,37^ and level of education.^37^ In a ‘minority’ African-American trial, culturally/age specific self-management strategies (e.g., gaming) were identified as a facilitator.^45^
- A facilitator that occurred frequently in studies involving South Asians across both majority and minority settings was providing culturally and linguistically appropriate educational materials. Language barriers were not an issue for ‘minority’ African Americans.^31,36,37^
- Some barriers and facilitators were specific to one of the two ethnic groups or social context. For instance, facilitators for ‘majority’ South Asian trials included generic self-management strategies,^30–32^ e.g., use of PAAPs,^30^ written reinforcement,^31^ and practising preventative behaviour.^32^ One African-American trial observed that stressors (e.g., neighbourhood violence), interfered with generic self-management strategies such as relaxation and breathing exercises in adolescents.^45^ Similarly, three African American trials incorporated discussions of managing common stressors in daily African American lives as a facilitator, because this allowed individuals to focus on asthma.^42,45,46^ Another African-American trial identified social support as a facilitator.^46^

## Discussion

### Main findings

We identified seventeen RCTs, most at unclear or high risk of bias, which tested asthma self-management interventions for South Asian or African-American communities. Education was a component of all interventions, but content, mode of delivery and additional strategies varied.^30–46^ Only two interventions were culturally targeted,^31,37^ in contrast to 15 culturally modified interventions,^30,32–36,38–46^ and no culturally tailored interventions. Trials based in South Asian countries,^30–33^ appeared to be more effective than those delivered to minority populations (for both South Asians and African Americans),^34–36,38–46^ though with the caveat that none of the ‘majority’ population trials were at low risk of bias and targeted populations were from tertiary care hospitals (in whom it may have been easier to demonstrate health benefits due to more severe asthma).^34,35,46^ Hence, it is unclear whether culture or minority-status of an ethnic group influences the variance in self-management outcomes. Education with on-going professional support was identified as a facilitator to asthma self-management in all groups.^31,32,37,39,44,45^ Other facilitators included focussing on individual learning styles in minority communities,^45^ culturally and linguistically appropriate education for minority and indigenous South Asians,^31,36,37^ generic self-management strategies in ‘majority’ South Asian communities,^30–32^ and strategies for dealing with stress and social support in African-American populations.^42,45,46^

### Interpretation of findings in relation to previously published literature

A previous systematic review^14^ concluded that a culturally targeted intervention^48^ (in line with the definitions of this review) was more effective than generic programmes in improving asthma outcomes, and revealed that most interventions were culturally modified. We found only two culturally targeted interventions,^31, 37^ suggesting that this recommendation has not been adopted, hence progress in this area of research has not advanced. This may be due to the expensive and lengthy nature of developing targeted or tailored interventions compared to the ease of adapting or re-testing modified interventions,^14, 17^ however, in the long-term culturally targeted or tailored interventions may be more cost-effective. Trials have typically considered ethnic groups as homogenous, e.g., they do not consider variation among smaller subcultural groups of South Asians or African Americans, or the influence of acculturation in minority communities, potentially important for designing interventions.^34–36, 38–46^ The two culturally targeted trials also included some modified characteristics, e.g., language adaptation for PAAPs, so the distinctions between culturally relevant interventions is not absolute. This is supported by a previous systematic review,^19^ which found interventions labelled as targeted or tailored also incorporated modified features, e.g., community/participatory approach to smoking cessation. It may be that modification of certain proven asthma self-management strategies, e.g., PAAPs, together with customising by culturally specific elements is an optimal approach.

Targeted trials customise the development of interventions to a cultural group rather than just adjusting the content. For instance, interventions developed collaboratively with target groups helped existing self-management strategies to be linguistically and culturally relevant.^7,16,31,37^ This can be further understood as aiming at deep structures, e.g., cultural beliefs, norms, lifestyles, environmental and social contexts, which aid receptivity of information and behaviour change. The Person-Based Approach^49^ to intervention development suggests that comprehension of user perspectives and contexts based on qualitative studies at every stage of development is central to customisation. In contrast, modifying surface structures to observable traits, e.g., language, ethnicity, food and clothing, may influence information processing but not behaviour change (a common characteristic of modified interventions).^23^ For instance, two ‘minority’ South Asian trials modified interventions according to language with mostly ineffective outcomes, suggesting merely focussing on language modifications is insufficient for their needs.^35,36^ However, more rigorous trials are needed, as both targeted interventions had either high or unclear risk of bias.^31,37^

Similarly, some ‘majority’ South Asian interventions were modified from generic programmes rather than developed for their own community.^30,32,33^ For example, Ghosh et al.^32^ a trial from India, adapted self-management strategies from an intervention from Colorado, USA.^50,51^ Trials from diverse sociocultural contexts and different cultural groups demonstrate the potential pitfalls of extrapolating findings from one context and applying it to another.^16,20,21^ A possible explanation for ‘majority’ South Asian trials incorporating culturally modified strategies may be that international clinical guidelines for respiratory diseases,^30–32^ e.g., GINA,^6^ promote a generic model of self-management interventions with evidence and examples from high-income populations and recommendation of adaption to low or middle-income countries (LMICs).^27^ While remaining true to the core evidence-based features of supported self-management presented in guidelines, intervention developers also need to deliberate on the principles of cultural relevance to the targeted local community, rather than depending on translation.^52^ For LMICs, this may be challenging due to the lack of resources, training and manpower, as well as public health priorities and models of care focusing on communicable rather than long-term conditions.^27,28,53^ GINA guidelines acknowledge these difficulties, but do not offer specific guidance on providing targeted or tailored self-management;^54^ in contrast to the advice about cost-effective options for diagnosis and treatment in LMICs.^6,28^

Conceptualising culture with its interaction with context offers new avenues of comprehending the role of culture in health. Apart from better outcomes in ‘majority’ South Asian trials based in tertiary care settings compared to ‘minority’ communities,^30–33^ poor reporting with limited descriptions of SES,^30–33,37–39,42,44,45^ and diversity of trial settings,^34–41,43–46^ meant we were unable to draw conclusions about associations between outcomes and contextual data. This is an important point as variations in SES within a culture has been suggested to determine health outcomes, e.g., restrictions in accessing services.^29^ In LMICs such as India, tertiary care may currently be the only practical setting for delivering asthma self-management interventions due to lack of community-based clinical and research expertise, as well as social and financial barriers that result in under-diagnosis, under-treatment and limited treatment availability. In the absence of adequately resourced primary care, it is common for individuals in these populations (particularly for children) to only access healthcare during exacerbations, rather than receiving preventative care.^28,53^

### Strengths and limitations of this study

To our knowledge, this review is one of few studies analysing the effectiveness of South Asian or African-American asthma self-management interventions. By identifying barriers and facilitators across two different ethnic groups and sociocultural contexts, our review can inform the customisation of interventions.^21,32,35^ We included seventeen trials, though the exclusion criteria of requiring separate outcome data for the specific groups of interest may have restricted the number of articles included in the final analysis; identification of more culturally targeted and even some tailored trials would have been informative. Limited resources precluded duplicate selection of papers, but we undertook a ten percent reliability check of the selection process. Risk of bias assessment was duplicated and data extraction was fully checked by a second reviewer.

Further, limited descriptions of the studies made it difficult to know how the interventions were developed or on what they were based on, particularly in the ‘majority’ South Asian trials.^30,32,33^ In addition, few authors responded to our request for further information. This meant that one of the targeted trials was excluded from the harvest plot analysis because data on between group differences were missing.^37^ Additionally, some harvest plot decisions relied upon sub-group analyses, which reduce study power and thereby could have increased the potential for null findings.^34,36,43^ However, primary outcomes were prioritised and, for clarity, inconsistent findings were indicated by hatched bars to limit over interpretation.^35,39^ Subjectivity in assessing the outcomes for the harvest plot was minimised by specifying predefined criteria that were replicable, and all the judgements were checked by at least two reviewers. Additionally, even though harvest plots are a good technique of illustrating heterogeneous findings and can be personalised to the requirements of the review, they may neglect some important outcomes that cannot be reported in the plots and overemphasise others.^4,55^

### Conclusions and implications for future research, policy and practice

Asthma self-management interventions delivered in South Asian and African-American minority communities were less effective than interventions delivered in indigenous populations in South Asia, though the design/conduct of the latter studies meant that they were at greater risk of bias. Additionally, most trials from India are not designing interventions to their community, instead they are following guideline recommendations from studies in high-income countries. Studies that improve understanding of sociocultural contexts, allow a deeper appreciation of customising interventions and how to prevent inequalities in self-management behaviour, both are needed to inform international asthma guidelines. Targeted or tailored intervention development does not exclusively include collaboratively developed components customised to beliefs and needs of the target ethnic group, but may also include adaption of existing resources. Intergroup subcultural heterogeneities, cultural changes over generations (due to acculturation) and individual learning styles, add to the complexity of self-management behaviour and all need to be explored further. Rigorous trials of culturally targeted or tailored interventions are needed. Moreover, there needs to be standard recommendations on how trials verify participant ethnicity/culture, as only three ‘minority’ South Asian trials defined ethnicity according to self-identification or language spoken and culture was not considered and/or perceived to be synonymous to ethnicity.^34,35,37^

## Methods

The review protocol is registered with the PROSPERO database (registration number CRD42015020174). We followed the procedures described in the Cochrane handbook for systematic review of interventions.^56^

### Search strategy

Our key search terms were ‘asthma’ ‘AND’ ‘self-management’ ‘AND’ ‘population’ (including terms for South Asian and Black communities as summarised in Table 4 (detailed in Supplementary Appendix 1). We searched for RCTs on eight electronic databases *(Medline, EMBASE, Web of Science, PsycINFO, Scopus, Elsevier Science Direct, Cochrane Library including Cochrane Airways Group Register of Trials and Google Scholar)*, three research registers in [February 2015] *(PROSPERO, The University of York’s Centre for Reviews and Dissemination, and the Clinical Trials Database)*, manually searched relevant journals (*Patient Education and Counselling*, *Health Psychology* and *Ethnicity and Health*), and searched reference lists of identified systematic reviews. The search was not confined by publication year or language.

**Table 4:** Search strategy terms

Asthma	Self-management	Population search
Asthma	Self management OR asthma control OR self care	South Asians
	Barriers OR facilitators	Bengali OR Bangladeshi OR Bangladesh
	Beliefs OR attitudes	Indian OR India
	Knowledge OR asthma education	Pakistani OR Pakistan
		Black OR African OR Afro Caribbean
		Ethnic OR ethnicity

### Inclusion and exclusion criteria

We included RCTs evaluating self-management interventions delivered to South Asian or Black asthma patients, the parents/carers of children with asthma, lay or healthcare professionals who care for people with asthma from these communities. The search included populations of all ages and in any country. Black African Americans, were included because they are from another well-studied minority population, with experience of socioeconomic deprivation, and our scoping of literature suggested there was a relatively large evidence base. Outcomes of interest were clinical (e.g., unscheduled care and asthma control),^57^ process, behavioural (e.g., knowledge and medicine adherence). We excluded studies that did not specify their population (e.g., trials using broad terms when describing their population such as ‘West Indians’ and ‘Asians’), and trials of multiple ethnic populations that did not provide separate asthma outcome data for the ethnic groups of interest (see Fig. 1; The PICO strategy is summarised in Table 5).

**Table 5:** PICO search strategy

PICO	Criteria
Population	South Asian communities (Indian, Pakistani, Bangladeshi etc.), or Black populations (African, Caribbean or Other) asthma patients, their parents/carers, healthcare or lay professionals. The search considered all population ages and countries
Intervention	Asthma self-management interventions in any healthcare, community or remote settings. We used the self-management definition of the US Institute of Medicine: *“The tasks that individuals must undertake to live with one or more chronic conditions. These tasks include having the confidence to deal with medical management, role management and emotional management of their conditions”*^60^
Comparator	Asthma patients, parents/carers of children with asthma, healthcare or lay professionals supporting asthma patients, who did not receive asthma self-management intervention
Outcomes	Outcomes of interest were:1. Clinical outcomes: (i) current asthma control was defined as the degree to which different asthma manifestations were reduced/eliminated by treatment. Here, main categories include clinical-asthma control level (ii) future risk of adverse events and unscheduled healthcare utilisation. All clinical outcomes are aligned with the American Thoracic Society/European Respiratory Society Task Force standardised definitions^57^2. Process outcomes: any outcome that occurred because of certain steps in a process, e.g., knowledge and self-efficacy3. Behavioural outcomes: outcomes related to behaviour, e.g., medicine adherence and inhaler technique
Exclusion	1. All studies that did not explicitly specify population were excluded e.g., trials that did not provide details on which ethnic group they are referring to when they used broad terms such as ‘West Indians’ or ‘Asians’2. Studies of multiple ethnic populations that did not provide outcome data separately for the South Asian and the Black ethnic groups or subgroups were excluded3. Trials studying multiple illnesses but did not provide separate outcome data for asthma were excluded

### Study selection

A PRISMA diagram was used to report the number of studies identified, the screening process and the final list of included studies (see Fig. 1). All titles, abstracts and full texts were screened by one reviewer (S.A.), and a random 10% by two other reviewers (L.S., H.P.). Disagreements were resolved by discussion and the inclusion/exclusion criteria clarified as necessary.

### Data extraction and risk of bias

A standardised Cochrane data extraction sheet was modified for this study.^58^ All data extraction was completed by one reviewer (S.A.) and independently checked by a second reviewer (K.H.). Discrepancies were resolved by discussions between reviewers and the wider team (L.S., H.P.), until consensus was achieved. Trial authors were contacted by email to clarify any missing, unclear or additional data required. If contact with the author failed, the uncertainty was noted on the data extraction form. The Cochrane EPOC Risk of Bias Assessment Checklist,^59^ was used to evaluate bias in included studies. This was independently coded by two researchers (S.A., K.H.), and any discrepancies were resolved by another researcher (L.S.).

### Analysis

We anticipated that studies would be too heterogeneous for meta-analysis, and, therefore, used a narrative synthesis, illustrating key findings on trial effectiveness with a harvest plot.^55^ Harvest plots allow visual representation of the findings of a narrative synthesis (comparable to Forrest plots in a meta-analysis), facilitating comparison across studies.^55^ They enable identification of interesting patterns among varying outcomes, and may highlight the strongest or most inconsistent evidence, areas of possible concern, and gaps in the research. If there were various outcomes in one category (e.g., the asthma control category might include symptom scores, symptom-free days, or days off work/school with a range of significant and non-significant results), the overarching outcome was determined according to predefined criteria (see note to Table 3), applied and agreed by three researchers (S.A., H.P. and/or L.S.).^55^ Sizes of lines and colour hatchings were used to illustrate features of the trial according to a defined convention (see summary in footnote to Fig. 2 and detailed description in Table 3). Barriers and facilitators were identified from data and/or interpretations of study authors.

### Data availability

All included papers are published; no further data are available. Requests for further information should be addressed to the corresponding author.

### Disclaimer

The views expressed are those of the author(s) and not necessarily those of the NHS, the NIHR or the Department of Health.

## Electronic supplementary material

Detailed Search Strategy
