# Supplementary material for: Interventions to enhance the adoption of asthma self-management behaviour in the South Asian and African American population: a systematic review
Source: NPJ Prim Care Respir Med. 2018 Feb 15;28:5. doi: 10.1038/s41533-017-0070-6 (PMC5814446; doi:10.1038/s41533-017-0070-6)
Supplement: Supplementary file 1 — Detailed Search Strategy [file 41533_2017_70_MOESM1_ESM.docx]

### Supplementary Appendix 1. Detailed Search Strategy

### *Basic search strategy:* Asthma search term AND Self-management terms AND Population search terms NOT Genetics NOT Qualitative OR Observational OR Cross-sectional OR Case control OR Cohort study (FILTERS: Humans; English)

### All searches in (title/abstract)

### Exemplar of search strategy on Medline database (Date searched, 18/02/15)

| Search | Query | Items found |
| --- | --- | --- |
| **#1** | Asthma | 148124 |
| **#2** | (Self management OR Self care OR Asthma control OR Barriers OR Facilitators OR Beliefs OR Attitudes OR Knowledge OR Asthma education) | 1607427 |
| **#3** | South Asians | 4385 |
| **#4** | (Bengali OR Bangladeshi OR Bangladesh OR Indian OR India OR Pakistani OR Pakistan) | 462725 |
| **#5** | Black | 159599 |
| **#6** | (African OR Afro Caribbean) | 143933 |
| **#7** | (Ethnic OR Ethnicity) | 243317 |
| **#8** | (Asthma) AND ((Self management OR Self care OR Asthma control OR Barriers OR Facilitators OR Beliefs OR Attitudes OR Knowledge OR Asthma education)) | 40018 |
| **#9** | ((((South Asians) OR ((Bengali OR Bangladeshi OR Bangladesh OR Indian OR India OR Pakistani OR Pakistan))) OR Black) OR ((African OR Afro Caribbean))) OR ((Ethnic OR Ethnicity)) | 849047 |
| **#10** | (((Asthma) AND ((Self management OR Self care OR Asthma control OR Barriers OR Facilitators OR Beliefs OR Attitudes OR Knowledge OR Asthma education)))) AND (((((South Asians) OR ((Bengali OR Bangladeshi OR Bangladesh OR Indian OR India OR Pakistani OR Pakistan))) OR Black) OR ((African OR Afro Caribbean))) OR ((Ethnic OR Ethnicity))) | 2706 |
| **#11** | Genetics | 2769758 |
| **#12** | (((((Asthma) AND ((Self management OR Self care OR Asthma control OR Barriers OR Facilitators OR Beliefs OR Attitudes OR Knowledge OR Asthma education)))) AND (((((South Asians) OR ((Bengali OR Bangladeshi OR Bangladesh OR Indian OR India OR Pakistani OR Pakistan))) OR Black) OR ((African OR Afro Caribbean))) OR ((Ethnic OR Ethnicity))))) NOT Genetics | 2366 |
| **#13** | (Qualitative OR Observational OR Cross-sectional OR Case control OR Cohort Study) | 1128116 |
| **#14** | (((((((Asthma) AND ((Self management OR Self care OR Asthma control OR Barriers OR Facilitators OR Beliefs OR Attitudes OR Knowledge OR Asthma education)))) AND (((((South Asians) OR ((Bengali OR Bangladeshi OR Bangladesh OR Indian OR India OR Pakistani OR Pakistan))) OR Black) OR ((African OR Afro Caribbean))) OR ((Ethnic OR Ethnicity))))) NOT Genetics)) NOT ((Qualitative OR Observational OR Cross-sectional OR Case control OR Cohort study)) | 1622 |
| **#15** | (((((((Asthma) AND ((Self management OR Self care OR Asthma control OR Barriers OR Facilitators OR Beliefs OR Attitudes OR Knowledge OR Asthma education)))) AND (((((South Asians) OR ((Bengali OR Bangladeshi OR Bangladesh OR Indian OR India OR Pakistani OR Pakistan))) OR Black) OR ((African OR Afro Caribbean))) OR ((Ethnic OR Ethnicity))))) NOT Genetics)) NOT ((Qualitative OR Observational OR Cross-sectional OR Case control OR Cohort study)) Filters: Humans | 1359 |
| **#16** | (((((((Asthma) AND ((Self management OR Self care OR Asthma control OR Barriers OR Facilitators OR Beliefs OR Attitudes OR Knowledge OR Asthma education)))) AND (((((South Asians) OR ((Bengali OR Bangladeshi OR Bangladesh OR Indian OR India OR Pakistani OR Pakistan))) OR Black) OR ((African OR Afro Caribbean))) OR ((Ethnic OR Ethnicity))))) NOT Genetics)) NOT ((Qualitative OR Observational OR Cross-sectional OR Case control OR Cohort study)) Filters: Humans; English | 1339 |
